# Novel Agonist Bioisosteres and Common Structure-Activity Relationships for The Orphan G Protein-Coupled Receptor GPR139

**DOI:** 10.1038/srep36681

**Published:** 2016-11-10

**Authors:** Mohamed A. Shehata, Anne C. Nøhr, Delphine Lissa, Christoph Bisig, Vignir Isberg, Kirsten B. Andersen, Kasper Harpsøe, Fredrik Björkling, Hans Bräuner-Osborne, David E. Gloriam

**Affiliations:** 1 Department of Drug Design and Pharmacology, Faculty of Health and Medical Sciences, University of Copenhagen, Universitetsparken 2, 2100 Copenhagen, Denmark; 2 Department of Neurodegeneration 1, H. Lundbeck A/S, Ottiliavej 9, 2500 Valby, Denmark

## Abstract

GPR139 is an orphan class A G protein-coupled receptor found mainly in the central nervous system. It has its highest expression levels in the hypothalamus and striatum, regions regulating metabolism and locomotion, respectively, and has therefore been suggested as a potential target for obesity and Parkinson’s disease. The two aromatic amino acids _L_-Trp and _L_-Phe have been proposed as putative endogenous agonists, and three structurally related benzohydrazide, glycine benzamide, and benzotriazine surrogate agonist series have been published. Herein, we assayed 158 new analogues selected from a pharmacophore model, and identified 12 new GPR139 agonists, containing previously untested bioisosteres. Furthermore, we present the first combined structure-activity relationships, and a refined pharmacophore model to serve as a rationale for future ligand identification and optimization.

G protein-coupled receptors (GPCRs) constitute the largest family of human membrane proteins^1^. GPCRs induce cellular signalling upon activation by a range of endogenous ligand types, including neurotransmitters, hormones, nutrients, peptides, and ions^2^. Approximately 1/3 of all marketed drugs act on GPCRs^3^. While many GPCRs are well-characterized there are still more than 120 non-olfactory orphan receptors with unknown endogenous ligand(s) and/or function^4^.

GPR139 is an orphan receptor identified from bioinformatics analysis of the human genome^5^. It has been shown to have a high mRNA expression in the brain, particularly in the striatum and hypothalamus^6^,^7^,^8^ – suggesting that GPR139 could be involved in movement control^7^,^8^,^9^ and/or the regulation of food intake/metabolism^6^,^10^. GPR139 is thus a potential target for the treatment of Parkinson’s disease, obesity, eating disorders, and/or diabetes. The main signal transduction pathway of GPR139 is still not established, as signalling through G_q_^6^,^10^,^11^,^12^, G_s_^9^, G_i_^8^ and G_12/13_^7^ have all been demonstrated. This ambiguity could be caused by a number of underlying reasons, including lack of studies of cells endogenously expressing the receptor, uncertainty about the endogenous ligand, the use of different cell lines as expression systems, and/or assay variations.

We have previously shown, that aromatic dipeptides and the endogenous _L_-amino acids tryptophan (_**L**_**-Trp**) and phenylalanine (_**L**_**-Phe**) (Fig. 1) can activate GPR139^10^ (EC_50_ of 220, and 320 μM, respectively). In addition, Liu *et al*. recently reported^6^,^13^ that _**L**_**-Trp** and _**L**_**-Phe** stimulate [^35^S]GTPγS binding in membranes from COS7 cells (EC_50_ of 26 *μ*M and 31 *μ*M, respectively), a concentration-dependent calcium response in HEK293 cells (EC_50_ of 49 *μ*M, and 60 *μ*M, respectively), and calcium mobilization in HEK293 (EC_50_ of 287 *μ*M and 411 *μ*M, respectively). This shows that these two aromatic amino acids activate GPR139, with EC_50_ values in the 30–300 *μ*M range. However, it cannot be excluded that the physiological activation of GPR139 is mediated by another more potent endogenous ligand.

Surrogate small molecule ligands for GPR139 have been reported by Hu *et al*.^9^, Shi *et al*. from Lundbeck A/S^11^, Dvorak *et al*. from Jansen R&D^13^,^14^, and Hitchcock *et al*. from Takeda Pharmaceutical Company Limited^15^. The most potent surrogate agonists are compounds **1a** (EC_50_ = 39 nM), **7c** (EC_50_ = 16 nM), and **39** (EC_50_ = 6 nM), from Shi *et al*., Dvorak *et al*. and Hitchcock *et al*., respectively (Fig. 1 and Tables 1, 2 and 3). Herein, we have screened our pharmacophore model^10^ against ~6 million drug-like compounds to identify more diverse agonist analogues, and conducted a joint structure-activity relationship (SAR) study together with the three published series.

## Results

### New agonist ligands and bioisosteres

**Figure 2:**
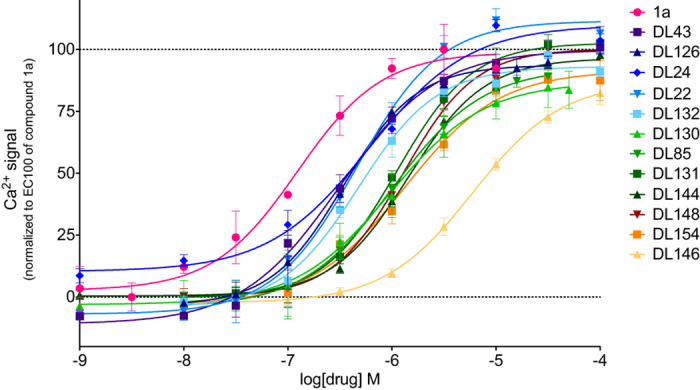
Concentration-response curves of the new GPR139 agonists measured on the NOVO-star using the Fluo-4 Ca^2+^-assay. The responses are normalized to EC_100_ of **1a** (8 μM) and are ordered by their potency (colour-scale: dark-blue > green > yellow). The graphs are one representative concentration-response curves out of at least three independent experiments performed in triplicates. Data points are means ± SD.

Our search for GPR139 ligands featured an initial pharmacophore screening followed by three cycles of assaying in the Fluo-4 Ca^2+^-assay and analogue purchases, and covered in total 158 compounds (Supplementary Chart 1). This yielded 12 novel GPR139 agonists with efficacies similar to compound **1a** (EC_50_ = 39 nM) and potencies ranging from 364 nM to 4.7 μM (Fig. 2 and Table 4). These contain previously untested bioisosteres of the terminal aromatic systems (Table 5), as well as variations of the linker compared to the published reference agonists. The 12 novel agonists did not show activity in the CHO-M1 receptor cell line in the Fluo-4 Ca^2+^-assay, indicating specific interactions with the GPR139.

### Characterisation of the preferred signalling pathway

**Figure 3:**
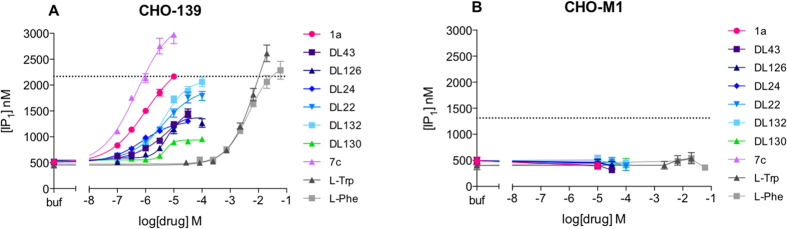
IP-One HTRF^®^ assay measuring G_q_ activation. Concentration-response curves of **1a**, **7c** (racemic), _**L**_**-Trp**, _**L**_**-Phe**, **DL43, DL126, DL24, DL22, DL132** and **DL130** on (**A**) CHO-139 and (**B**) CHO-M1 in the IP-One assay (final conc of LiCl = 20 mM). The dotted line represent the [IP_1_] response from (**A**) 10 μM **1a** and (**B**) 1 μM carbachol, which is an agonist for the muscarinic acethylcholine receptor M1 that activates the G_q_ coupled pathway. The graphs are means ± SEM based on three independent experiments performed in triplicates.

Due to the different signalling pathway reported in the literature^6^,^10^,^11^,^12^, we tested two of the most potent known surrogate agonists; **7c** and **1a**, the _**L**_**-Trp** and _**L**_**-Phe** and the submicromolar compounds (**DL43, DL126, DL24, DL22, DL132** and **DL130**) in the cAMP dynamic 2 assay (CisBio) and IP-One HTRF^®^ assay (CisBio) to check for biased signalling in the G_s_, G_i_ and G_q_ coupled assay. None of the tested compounds induced G_s_ response in CHO-139 or in CHO-M1 cells (Supplementary Fig. 1), which was included as a control. Furthermore none of the tested compounds were able to inhibit the cAMP response induced by 5 μM forskolin in the CHO-139 cells by activating G_i_, except _**L**_**-Trp**, which inhibits the 5 μM forskolin induced cAMP response by more than 10% in high concentrations, indicating G_i_ activation (Supplementary Fig. 2). However, this response was seen in both CHO-139 and CHO-M1 and is therefore an unspecific response. Hence, based on our data the compounds described herein activate GPR139 in CHO-k1 cells through the G_q_ pathway, which is shown with both the Fluo-4 Ca^2+^-assay (Fig. 2 and Table 4) and the IP-One assay (Fig. 3A and Table 6). Furthermore, we show that the compounds activate CHO-GPR139 specifically through GPR139, as no response is seen in CHO-M1 cells (Fig. 3B).

Overall there was a good correlation between potencies observed in the Fluo-4 Ca^2+^-assay and the IP-One assay with compounds generally being 10–40 fold more potent in the former. This has been observed previously and has been ascribed to spare receptors where only a small proportion of receptors need to be activated to elicit a full calcium response^16^. Interestingly, the DL-compounds tested in both assays behave as full agonists in the Fluo-4 Ca^2+^-assay but as partial agonists in the IP-One assay. Moreover, racemic **7c** and _**L**_**-Trp** behave as super-agonists compared to compound **1a** (Table 6). The IP-One assay is thus superior in differentiating the efficacy of the compounds.

### Collective SAR – A common motif of two terminal phenyl rings, R1 and R2, separated by a 6-atom linker

**Figure 4:**
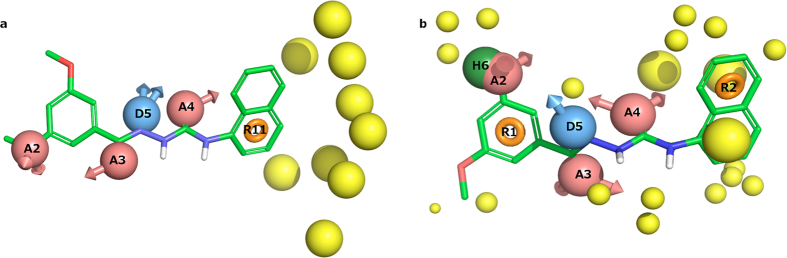
Comparison of the original and new pharmacophore models. (**a)** The original pharmacophore model used to identify the new agonists, and (**b)** the refined pharmacophore model based on the currently presented SAR analysis. Compound **1a** is shown with green carbons. Pharmacophore features are: Red: hydrogen bond acceptor, Blue: hydrogen bond donor, Green: hydrophobic element, Orange: aromatic system, and Yellow: exclusion volume (eliminates matching of substituents resulting in activity loss).

The lowest energy conformations of the three most potent published reference agonists **1a**, **7c** and **39** demonstrated a near perfect overlay of their terminal phenyl rings, herein designated **R1** and **R2** (Tables 1, 2 and 3), and a six atom linker with multiple polar groups (Supplementary Fig. 3). Below we present the first collective SAR analysis of the three series, and our new analogues, which are summarized in the form of a common pharmacophore model (Fig. 4b) that refines our original model used for the virtual screening by incorporating the three latter (new) series.

### Linker length

The linker in **1a** has a central nitrogen flanked by two amide groups (Table 1). **7c** and **39** differ by having a carbon in the third position, in effect making the central portion of the linker a glycine moiety, and by having a methylated aliphatic carbon in the 6^th^ position (Tables 2 and 3) instead of an aromatic carbon in **1a**. Our new agonists (Table 4) display variations of either carbon or nitrogen atoms in the positions 3, 5 and 6, specifically: CNC (**DL130–132**), NCC (**DL146**), NCN (**DL22** and **DL144**), and NNC (**DL148**).

**Figure 5:**
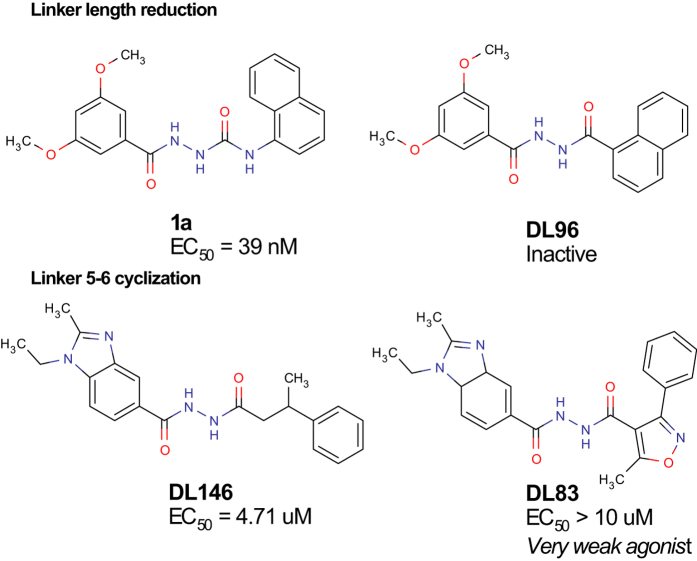
Chemical structures of compounds 1a, DL96, DL146, and DL83 showing that both linker reduction (top) and 5-6-cyclization (bottom) are unfavourable for the potency.

Removal of the 5-nitrogen (**DL96**) in **1a** resulted in loss of activity (Fig. 5), indicating that a reduction of the linker length cannot be tolerated. This is consistent with the presence of a shortened linker in many of the inactive analogues, such as **DL3**, **DL5–8**, **DL15**, **DL47**–**48**, **DL51**, **DL55**, **DL59**, **DL61**, **DL95**, and **DL153** (Supplementary Chart 1). However, **DL24,** in which the 5-position and **R2** are fused with a cyclohexyl, makes up an exception maintaining activity (EC_50_ = 431 nM) despite a shorter linker. Interestingly, **DL24** matched the pharmacophore elements well by adopting an atypical out-of-plane conformation between the acceptor (**A4**) and the aromatic (**R1**) pharmacophore elements (Supplementary Fig. 4 and Supplementary Chart 1). This may suggest that the cyclization rescues activity by locking the orientation of the **R2** phenyl, which is free to rotate in the closest inactive analogues, **DL6–8**. We also found that generally compounds with linkers longer than 6-atoms, has decreased activity such as compounds **73, 79** and **80** from Hitchcock *et al*.^15^ and our inactive ligands **DL50**, **DL78**, **DL84**, **DL86**, **DL98**, **DL104**, **DL105**, **DL113**, and **DL155** (Supplementary Chart 1).

### Linker hydrogen bond features

Removal of the 1-carbonyl oxygen atom (**1p** and **9** Dvorak *et al*.) results in loss of activity suggesting that it represents a critical hydrogen bond acceptor (feature **A3** in Fig. 4b). Methylation (**1t**) or carbon substitution (**1q**) of the 2-nitrogen reduces the potency to the micromolar range suggesting that a hydrogen bond donor in this position is important (feature **D5**). The 2-nitrogen in **39** lacks a hydrogen bond donor feature, however, **39** and the rest of the Hitchcock *et al*. set can perfectly fit the suggested pharmacophore model in a horizontally flipped manner (Supplementary Fig. 5) suggesting **D5** is still a fair-to-assume pharmacophore feature based on all other ligand sets. In contrast, replacement of the 3-nitrogen with carbon (**1r**, and all **7a–q**) has little effect on potency. Removal of the 4-carbonyl oxygen atom (**8** – Dvorak *et al*.) results in loss of activity suggesting that it represents a critical hydrogen bond acceptor (feature **A4**). At the 5-position, nitrogen to carbon substitution (**1a** to **1s**) has a marginal (2-fold) effect. The new agonists **DL131** and **DL132** corroborate this as they maintain the same three moieties in positions 1, 2 and 4, while those in positions 3 and 5 are both absent.

### Linker conformational restraints

The series by Hitchcock *et al*.^15^ has a constrained linker at the 2-position via a 4-oxo-3,4dihydrobenztriazene ring. This might be a beneficial strategy to lock the aromatic ring and/or the linker 1–2 atoms in a favourable conformation. This is proved by the generally improved potencies in the Hitchcock analogues (e.g. **39** vs. **1a** and **7c**). In the series by Dvorak *et al*.^13^,^14^, the 3-carbon has been substituted to change the central Gly residue into Ala, Ser, and Phe (**10–13**). The *S-*(**11**), but not *R-*methylation (**10**), strongly reduced the potency (compared to **7h**). In the Hitchcock *et al*.^15^ set, the same effect is seen in compounds **50** and **51** (compared to **46**). Furthermore, even with the favourable enantiomer, hydroxymethyl substitution (**12**) also leads to a significantly reduced potency. Thus, although part of the effect could be mediated by the action of the two flanking sides of the ligands, there is no room for bulk around the 3-position and exclusion volumes were placed accordingly (Fig. 4b). Moreover, the 1- (**1p** and **8**) and 4-carbonyl (**9**) oxygen atom removal that abrogated activity may have done so partly because of the loss of the *sp2* atom configuration, in addition to the loss of the hydrogen bond acceptor character.

**DL132** (EC_50_ = 505 nM) and **DL83** (EC_50_ > 10 μM – *very weak agonist*) are cyclized between the 5- and 6-positions. This has not been tested before, and it would be intriguing to investigate this cyclization on the two published series. As shown below, the 6-position is often fused with the **R2** phenyl, but can also be aliphatic and methylated (**7**–**13**). Of note, both the 6-position (*S*) and (*R*) enantiomers of **7c**^13^,^14^ as well as compounds **5** and **6** in the Hitchcock *et al*. set^15^ are potent, suggesting no preferred geometry in the binding pocket around this position.

### R1 substitution and bioisosteres

Removal of one of the 3,5-diMeO groups reduces potency by ~26-fold in the Shi *et al*. series (**1f** vs. **1a**), but has no effect in the series by Dvorak *et al*. (**7h** vs. **7q**), which contains several additional equipotent single or double substituted analogues: methyl (**7e**), trifluoromethyl (**7i**), methoxy (**7h**, **7q**) trifluoromethoxy (**7j**), cyanonitrile (**7f**), and several halogens (F: **7g**; Cl/F: **7o**, Br/Cl: **7p**). This is also seen in the Hitchcock *et al*.^15^ series where R1 has a 3-methoxy (e.g. **18** and **60**), methyl (e.g. **28**), triflouromethyl (e.g. **43**), or several halogens (e.g. F: **14**; Cl: **19**). Together, this suggests the combined 3,5-substitution is not needed to maintain potency. Interestingly, the alternative pattern of 2,3- (**7k**) and 2,5-dichloro- substitution (**7m**) is equipotent to the 3,5-pattern (**7n**), but unlike the single 3-chloro (**7c**), the 2-chlorosubstituent (**7b**) alone displays a slightly reduced potency (~3-fold). This is in line with the series by Shi *et al*.^11^ and Hitchcock *et al*.^15^ in which a single methoxy in the 2-position (**1c,** and **3**, respectively) is (~16- and 5-fold) less potent than the 3,5-diMeO reference (1a, and 39). This SAR at the 2-position is shared by the 4-position (**35**, **37** and **1j–m**): A third methoxy group into the 4-position (**1h**) of **1a** shows that the substituent can be accommodated, however the single substituent alone (methyl: **35**, methoxy: **37**, fluoro: **1g**, chloro: **7d**, and phenoxy: **1i**) is not enough to maintain very high activity. Collectively, this shows that the 3-position is essential and can hold either a hydrophobic/halogen or acceptor element (H6/A2), whereas the 2,4, and 5-substitutions are optional (**1f**, and **36** being exceptions).

With regard to the size of **R1** substituents, ethyloxy-substitution in the 2- (**1d**), and di-ethyl in the 3 and 5-positions (**1e**) abrogates and significantly reduces activity, respectively (represented as exclusion volumes placed on the third atoms in all of the positions 2,3, and 5). However, smaller substituents on the 2-position (**39**) can be accommodated in the Takeda set and none of the added exclusion volumes are in contradiction with any of those substituents (Supplementary Fig. 4). In contrast, the 4-position can accommodate the large 1-ethyl-2-methyl-benzoimidazole bioisostere found in multiple active compounds (**DL43, DL85, DL164** and **DL148**). Finally, all active surrogate agonists present an **R1** terminal aromatic ring in our series, Shi *et al*.^11^, Dvorak *et al*.^13^,^14^, and Hitchcock *et al*.^15^, which likely contributes to the potency (feature **R1** in Fig. 4b).

The new agonists span three 3,5-diMeO/3-Cl/ 2-Me-phenyl bioisosteres: 1-ethyl-2-methyl-benzoimidazole, benzo[*d*][1,3]dioxole, and 1H-benzo[*d*]imidazole (Table 5). The two most potent new agonists share the distal naphthyl (**R2**) moiety of **1a**, while the 3,5-diMeO phenyl (**R1**) is replaced with 1-ethyl-2-methyl-benzoimidazole (**DL43**) or benzo[*d*][1,3]dioxole (**DL126**). Their potencies are ~10-fold lower than that of **1a** (in the Fluo-4 Ca^2+^-assay) but part of this could be attributed to a nitrogen-to-carbon substitution at linker position 5 (led to ~2.4-fold reduction in **1s** vs. **1a**). Thus, these two bioisosteres are likely to be nearly equipotent to the reference 3,5-diMeO- and 3-Cl-phenyl moieties. Moreover, our **R1** bioisosteres have polar surface area (PSA) and solubility (QPlogS, QPlogP_o/w_) values comparable to the parent moieties in **1a, 7c**, and **39** (Table 5).

### R2 substitution and bioisosteres

The series by Dvorak *et al*.^13^,^14^ and Hitchcock *et al*.^15^ showed that the **1a** naphthyl can be replaced by a benzyl-ethyl, i.e. the distal of the two fused aromatic rings is sufficient to maintain the high potency (feature R2 in Fig. 4 was moved accordingly). Our data showed that replacing the terminal phenyl ring **R2** with a hetero-aromatic ring system, e.g. **DL8, DL20 and DL40** (Supplementary Chart 1), led to a complete loss of activity. In the Shi *et al*. series, substitutions in the aromatic ring closest to the linker, could be tolerated in the 2-position (methyl: **1l**), but not any of the 4-(bromo: **1j**, cyanonitrile: **1k**, chloro: **1m**), 5-(bromo: **1n**), or 7-positions (methoxy: **1o**). Furthermore, inactivity was also observed for several compounds with bulky substituents: di-phenylmethyl (**DL94**, **DL80**), cyclopentyloxyphenyl (**DL134**), and pyridinylmethylmorpholine (**DL135**). However, the large **DL85** and **DL148** make up exceptions able to exhibit activity (EC_50_ = 1.02 μM and 4.7 μM, respectively). In the series by Hitchcock *et al*. small substitutions on **R2** seems tolerable and sometimes even favourable (**1** vs. **5**). Taken together, this suggests a tight fit to the receptor-binding cavity in some positions around **R2** (exclusion volumes are added at positions 4, 5 and 7 accordingly).

The new agonists span five **1a** naphthyl/**7c, 39** benzyl bioisosteres: 5,6,7,8-tetrahydronaphthyl, 1,2,3,4-tetrahydroquinoline, thiochromane, naphtha[2,1-b]furan, and 2,3-dihydro-1H-indene (Table 5). All bioisosteres are fused ring systems with a non-aromatic first ring, except the naphtha[2,1-b]furan, which notably has three fused aromatic rings. Similar to **R1**, our **R2** bioisosteres have polar surface area and solubility values comparable to the parent moieties in **1a, 7c**, and **39** (Table 5).

### Common pharmacophore model

#### Pharmacophore composition

The above collective SAR was used to construct the updated pharmacophore model in Fig. 4B, and the detailed mapping of analogues into features is listed in the last columns of Tables 1–3. The pharmacophore contains two terminal aromatic features (R1 and R2) separated by a linker containing two hydrogen bond acceptors (A3 and A4), and one hydrogen bond donor (D5). An additional dual hydrophobic/halogen or acceptor element, H6/A2, is placed at position 3 of R1. Finally, exclusion volumes (yellow) have been incorporated to block substituents rendering a reduction or loss of activity.

#### Pharmacophore Validation

**Figure 6:**
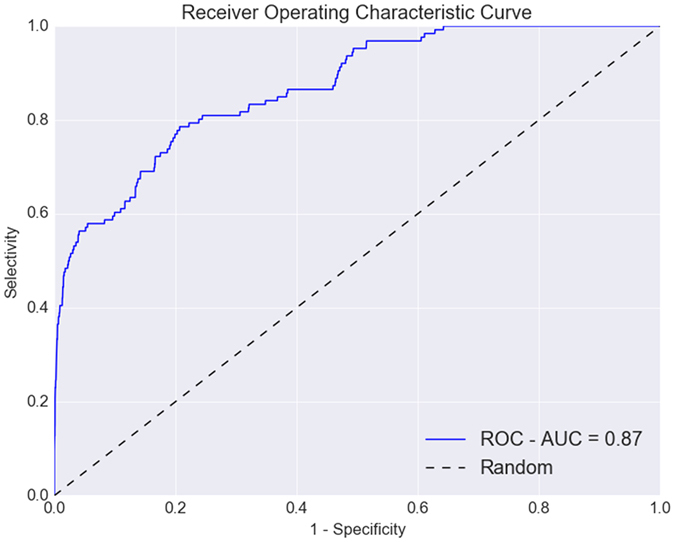
ROC – Plot of the pharmacophore matching of all published, new compounds, and set of DUD-E generated decoys^17^. The area under the curve is 0.87, and the top 1% includes 72% actives, and 80% of the actives are matched in the first 22% of the screening set, demonstrating a very high discrimination of the active from inactive compounds.

The updated pharmacophore model was matched against all 126 agonists described herein, and 6300 property-matched decoys generated by DUD-E^17^ (50 decoys/ligand). The pharmacophore matched all known agonists, including the new analogues described herein. A receiver-operating characteristic (ROC) analysis displayed an area under the curve of 0.87 (Fig. 6). The early enrichment (top 1% of matches) was 72% actives, and strikingly 80% of all actives were retrieved already in the first 22% of the entire set, displaying both a good selectivity and sensitivity of the pharmacophore model. Matching of the same set of compounds against our original pharmacophore model (only based on the set by Shi *et al*.^11^) displayed a significant improvement (Supplementary Fig. 7). Our refined pharmacophore model successfully filtered out inactive analogues due to the added exclusion volumes, which did not hinder matching of the new series by Hitchcock *et al*.^15^ (Supplementary Fig. 6). Collectively, this suggests that the new pharmacophore model can be used to find potentially active analogues in future prospective screenings.

## Discussion

The new bioisosteres and the common SAR analysis present features that could be useful in future medicinal chemistry and optimisation studies. For example, a **7c** naphthyl to benzyl substitution would be an interesting combination of two of the three published series. From the new linker variants, cyclization of the linker 5- and 6-positions, as in **DL132**, on the three published series could explore the conformational restraining of the **R2**-naphthyl and benzyl systems. Additionally, the necessity of the linker 1-position carbonyl oxygen’s lone pair as a hydrogen bond acceptor is clear, but a carbonyl bioisostere, such as a spiro-oxetane, should provide the same electronic function while also testing a slight increase in steric bulk in a region devoid of exclusion volumes. On the other hand, constraining the linker 2-position via cyclization with a 4-oxo-3,4-dihydro-benzotriazene ring as in **39** and the rest of the Hitchcock series, showed a potential beneficial effect for the activity. Furthermore, some of the new bioisosteres might be able to give equivalent or close potencies if introduced to otherwise identical analogues of the reference ligands, while offering slightly modified solubility and polar surface areas (PSA) as described below.

As mentioned by Shi *et al*. it would be beneficial for brain penetration to reduce the polar surface area of GPR139 agonists^11^. Blood-brain barrier penetration is achieved when PSA is lower than 90 Å^2 ^18, and the PSA of **1a** is just short of this, 88.70 Å^2^. **1s** displays an improved PSA of 76.7 Å^2^, but failed to increase brain exposure due to low plasma levels^11^. In contrast, **7c** displays good cellular permeability (and no efflux potential), and a brain to plasma ration of 1.2^14^. Some of our bioisosteres could offer slightly more beneficial PSA values (Table 5). For **R1**, the PSA of 1-ethyl-2-methyl-benzoimidazole is slightly less than that of 3,5-diMeO-phenyl in **1a**, and the benzo[*d*][1,3]dioxole has a comparable value. For **R2**, 5,6,7,8-tetrahydronaphthyl, thiochromane, and 2,3-dihydro-1 *H*-inden have equivalent PSA values to the two reference systems.

The PSA must be balanced with the solubility estimated with recommended QPlogS^19^ values between −1 and −6 mol/dm^–3^; to achieve CNS penetration typically a QPlogP_o/w_ value below 3.5 is desired. The most soluble **R1** bioisosteres are: benzo[*d*][1,3]dioxole > 1H-benzo[*d*]imidazole = 3,5-diMeO-phenyl (**1a**) > **7c** 3-Cl-Phenyl, while 1-ethyl-2-methyl-benzoimidazole is comparable to **7c** 3-Cl-Phenyl. For **R2**, 1,2,3,4,tetrahydroquinoline, thiochromane, and 2,3-dihydro-1 *H*-inden have equivalent or better solubility than **1a**. However, the benzyl in **7c** remains the option with the lowest values.

## Conclusions

In the present study we identified 12 GPR139 agonists (EC_50_ = 364 nM to 4.7 μM) containing previously untested aromatic bioisosteres, as well as novel linker variants. Our compounds were incorporated in the first combined SAR study of the three most potent series published by Lundbeck A/S, Janssen R&D and Takeda Ltd. This SAR was used to suggest a refined common pharmacophore model, which was able to discriminate between active and inactive compounds. The two proposed endogenous agonists _**L**_**-Trp** and _**L**_**-Phe** overlay with **R1** and **R2**, respectively as well as additional linker functionalities (Supplementary Fig. 8) showing that they too are likely to be accommodated by the common pharmacophore and the same binding site. This study could serve to guide the future ligand identification and optimization efforts. Studies to characterize the pharmacology and function of GPR139, as well as to identify antagonist tool compounds, are ongoing.

## Methods

### Ligand preparation and conformational search

LigPrep^20^ was used for ligand preparation. Default QikProp^21^ settings were used to calculate PSA, QPlogS, and QPlogP_o/w_ values. Macromodel^22^ was employed to do the conformational analysis on the three potent ligands **1a**, **7c**, and **39** using default settings. This includes using the OPLS_3 force filed^23^, and the Monte Carlo approach for sampling the different conformations. The global energy non-collapsed conformation of the ligands was picked for further analysis and superposition. Hitchcock criteria^18^: Sum of nitrogen and oxygen atoms (N + O) < 5, ClogP – (N + O) > 0, PSA < 90 Å2, and MW < 450 were also employed to assess the bioisosteres.

### Pharmacophore screening

The screening database, eMolecules plus^24^ (~6 million drug-like compounds), was prepared with LigPrep^25^ to desalt, add hydrogen atoms and generate tautomers, stereoisomers (max 32) and 3D conformations (max 10 ring conformations). Epik and the OPLS 2005 force field^26^ were applied to generate charge states at pH: 7.0 ± 1.0^22^. LigFilter was used to remove structures with reactive functional groups. The Phase database was prepared with 100 maximum conformers, up to 10 conformations per rotatable bond, thorough conformational sampling, conformational variation of amide bonds and a maximum relative energy difference of 6.0 kcal/mol.

### Hit and analog purchases

A minimum of four matching pharmacophore^10^ elements was required and a preference was set for partial matches involving more sites. Hits were sorted by fitness score and clustered with Canvas^27^ to select diverse representative structures. After the first assaying round small structure-activity relationship analyses were conducted and the compounds sorted into lead ligand series. The selections of analogs were based on substructures drawn in MarvinSketch and queried in the eMolecules database loaded into Instant JChem (Marvin 5.12.3, 2014 and Instant JChem 6.2.0, 2014, ChemAxon, www.chemaxon.com). Compounds were purchased from Enamine (Kiev, Ukraine).

### Compounds and buffers

Compound **1a**^11^ was kindly provided by H. Lundbeck A/S, Denmark. Compound **7c** (Enamine no: Z31449867) was tested as a racemate, as the (S)-form described by Janssen *et al*. was not commercially available. All compounds were dissolved to 20 mM in DMSO (Sigma, D2650) and subsequently diluted in a HEPES buffer (HBSS (Invitrogen, 14025) supplemented with 20 mM HEPES + 1 mM MgCl_2_ + 1 mM CaCl_2_, pH = 7.4) to a final concentration 0.5% DMSO in the assay. The DMSO level was kept constant for all concentrations of all compounds. DMSO was confirmed not to have any activity by itself at this concentration^10^. _**L**_**-**Tryptophan (T0254) and _**L**_**-**Phenylalanine (P2126) were obtained from Sigma-Aldrich and dissolved directly in buffer.

### Cell lines

All compounds were tested on a CHO-k1 cell line stably expressing GPR139 (CHO-GPR139) kindly provided by H. Lundbeck A/S, Denmark, and also on a CHO-k1 cell line stably expressing the M1 receptor (CHO-M1) (The Missouri S&T cDNA Resource Center, #CEM100TN00) to check for specificity. The CHO-GPR139 was grown in Dulbecco’s modified eagle medium (DMEM) F12-Kaighn’s (Gibco, 21127) supplemented with 10% dialysed fetal bovine serum (Gibco, 26400, United States origin), 1% GlutaMAX^TM^-I (100X) (Gibco, 35050), and 100 units/mL penicillin and 100 μg/mL streptomycin (Gibco, 15140) and 1.0 mg/mL geneticin (Invitrogen, 1181103). CHO-M1 was grown in Ham’s F12 (Gibco, 21765) supplemented with 10% fetal bovine serum (Gibco, 10270, South America origin) +100 units/mL penicillin and 100 μg/mL streptomycin (Invitrogen, 15140–122) and 0,25 mg/mL geneticin (Life Technologies, 11811–031). Compound **1a** and carbamoylcholine chloride (Sigma-Aldrich, C4382) were used as positive controls in the two cell lines, respectively.

### Ca^2+^-Fluo-4 assay

30.000 cells/well were plated in black 96-well plates with flat clear bottoms (Corning, Falcon, 353219) and incubated overnight. The Fluo-4 dye loading solution (Invitrogen, F36206) was prepared according to the manufacturer’s instructions by dissolving it in HEPES buffer supplemented with 2.5 mM probenecid. 50 μL dye loading solution was added to each well. Cells were incubated with the Fluo-4 dye for 60 min at 37 °C, then washed with 100 μL HEPES buffer. 100 μL HEPES buffer supplemented with 2.5 mM probenecid was then added to each well and incubated in 10 minutes at 37 °C before measurement. 33 μL of the test compounds (4x concentrated) were added automatically after baseline measurements. Intracellular calcium change was recorded on a NOVOstar instrument (BMG Labtech) at 37 °C with an excitation filter of 485 nm and an emission filters 520 nm. Data originate from three independent experiments in triplicates.

### IP-One assay

The IP-One assay was performed as described in the work by Thomsen *et al*.^28^, with few modifications. Breifly, CHO-139 and CHO-M1 were detached and re-suspended to a concentration of 10 million cells/mL. 5 μL 2x concentrated compound (+40 mM LiCl) and 5 μL cell suspension (50,000 cells/well) was mixed. The plate was sealed and incubated at 37 °C for 1 hour. Next, 10 μL detection reagents (lysis buffer containing 2.5% Eu^3+^-anti-IP1 antibody and 2.5% IP1-d2) was added and the plate was incubated for 1 hour at room temperature. The plate was read on an Envision (PerkinElmer, Waltham, MA, USA). The time resolved-fluorescence resonance energy transfer (TR-FRET) 665 nm/615 nm ratio, which is inversely proportional to the inositol monophosphate (IP_1_) accumulation, was used to determine the concentration of the IP_1_ response.

### cAMP assay

The cAMP assay was performed as described in the work by Thomsen *et al*.^28^ with few modifications. Breifly, CHO-139 and CHO-M1 was detached and resuspended to a concentration of 1 million cells/mL. 5 μL 2x concentrated compound (for G_s_: +100 μM IBMX and for G_i_: +100 μM IBMX +10 μM forskolin (Fsk)) and 5 μL cell suspension (5,000 cells/well) was mixed. The plate was sealed and incubated at room temperature for 30 min. Next, 10 μL detection reagents (lysis buffer containing 2.5% Eu^3+^-anti-cAMP antibody and 2.5% cAMP-d2) was added and the plate was incubated for 1 hour at room temperature. The plate was read on an Envision (PerkinElmer, Waltham, MA, USA). The TR-FRET 665 nm/615 nm ratio, which is inversely proportional with the cAMP production, was used to determination the concentration of the cAMP response.

### Generation of a common pharmacophore model

Phase version 4.5^29^ was used to build the new pharmacophore model based on compounds from all GPR139 agonist series (Tables 1–4). Ligand conformations were generated with the thorough sampling option and hypotheses matching the variant AAADHRR (A hydrogen bond acceptor, D hydrogen bond donor, H hydrophobic group, and R aromatic ring) were taken into consideration. The EC_50_ cut-off was set to 1 μM and the hypothesis matching the defined elements was selected after scoring active and inactive compounds. Exclusion volumes were added to exclude inactive ligands volume sizes were defined based on the size of disfavoured substituents. 2D structures were drawn in MarvinSketch^30^ and 3D structures were visualized in PyMOL^31^.

## Additional Information

**How to cite this article**: Shehata, M. A. *et al*. Novel Agonist Bioisosteres and Common Structure-Activity Relationships for The Orphan G Protein-Coupled Receptor GPR139. *Sci. Rep*. **6**, 36681; doi: 10.1038/srep36681 (2016).

**Publisher’s note:** Springer Nature remains neutral with regard to jurisdictional claims in published maps and institutional affiliations.

## Supplementary Material

Supplementary Information

## Figures and Tables

**Figure 1:**
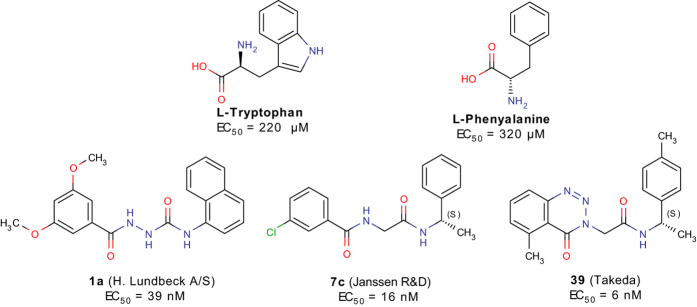
Chemical structure of the two endogenous amino acids _L_-Trp and _L_-Phe, and the most potent surrogate agonists 1a and 7c, and 39 from three series published by H. Lundbeck A/S^11^, Janssen R&D^13^,^14^, and Takeda^15^ respectively.

**Table 1:**
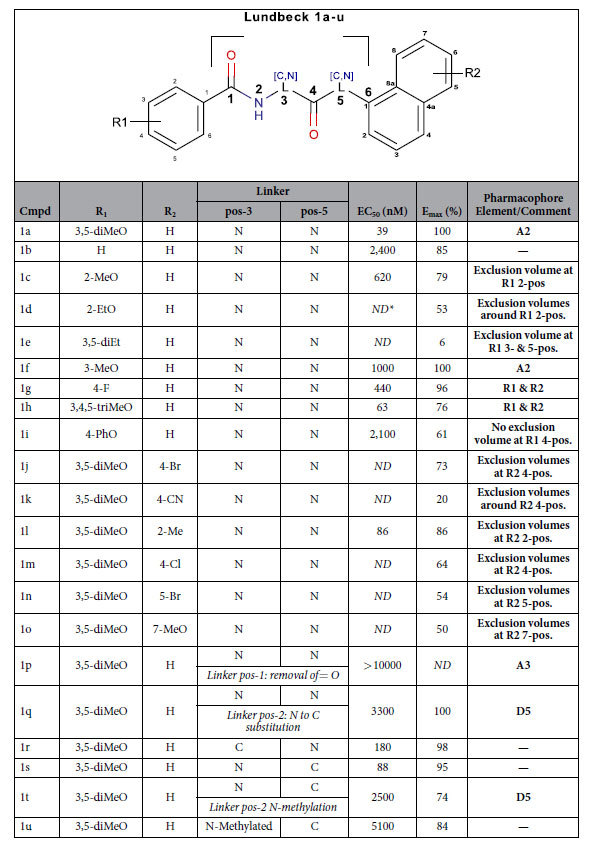
Chemical structures and potencies of compounds 1a-u.

Data from Shi *et al*.^11^. *ND*: not determined.

**Table 2:**
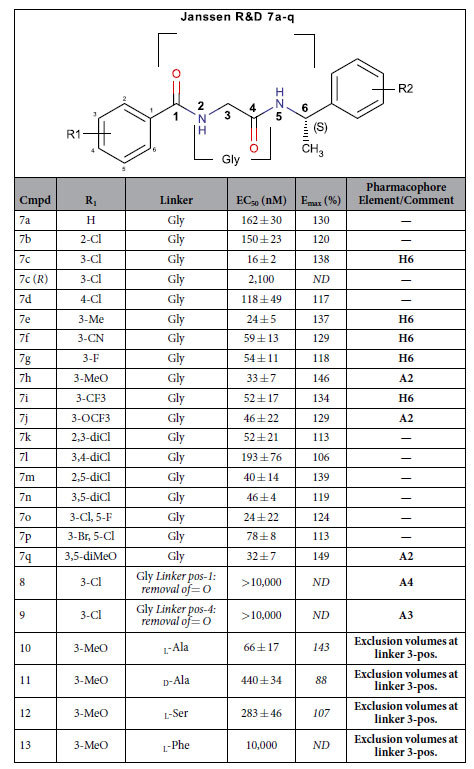
Chemical structures and potencies of compounds 7a–q.

Data from Dvorak *et al*.^13^,^14^. The maximum efficacy (**E**_**max**_) is relative to _**L**_**-Trp**. All data represent the mean of at least three different experiments. *ND*: not determined.

**Table 3:**
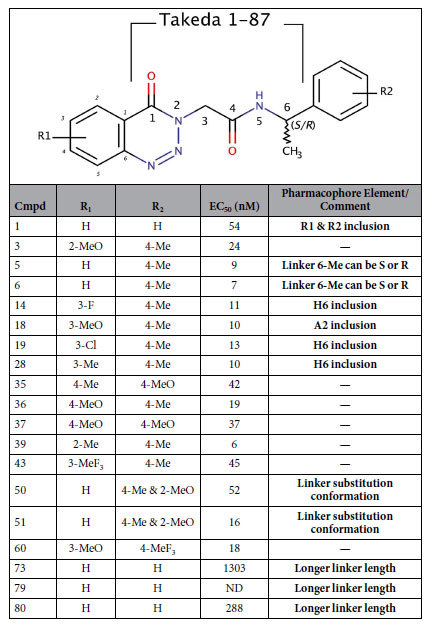
Chemical structures and potencies of selected compounds from Hitchcock *et al*.^15^ confirming the SAR analysis for GPR139 analogues.

**Table 4:**
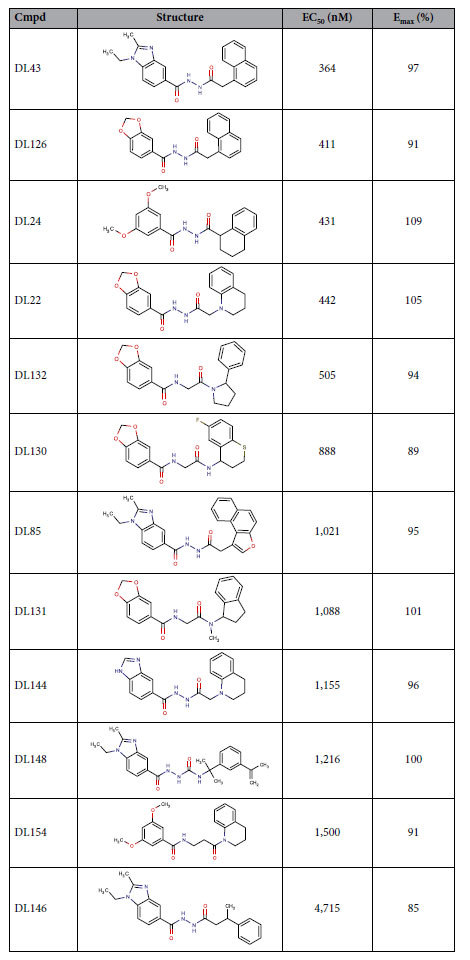
Structures, potencies, and efficacies of the 12 new agonists as recorded in the Fluo-4 Ca^2+^-assay.

Data represent the mean of at least three different experiments. Efficacies (**E**_**max**_) are relative to **1a**.

**Table 5:**
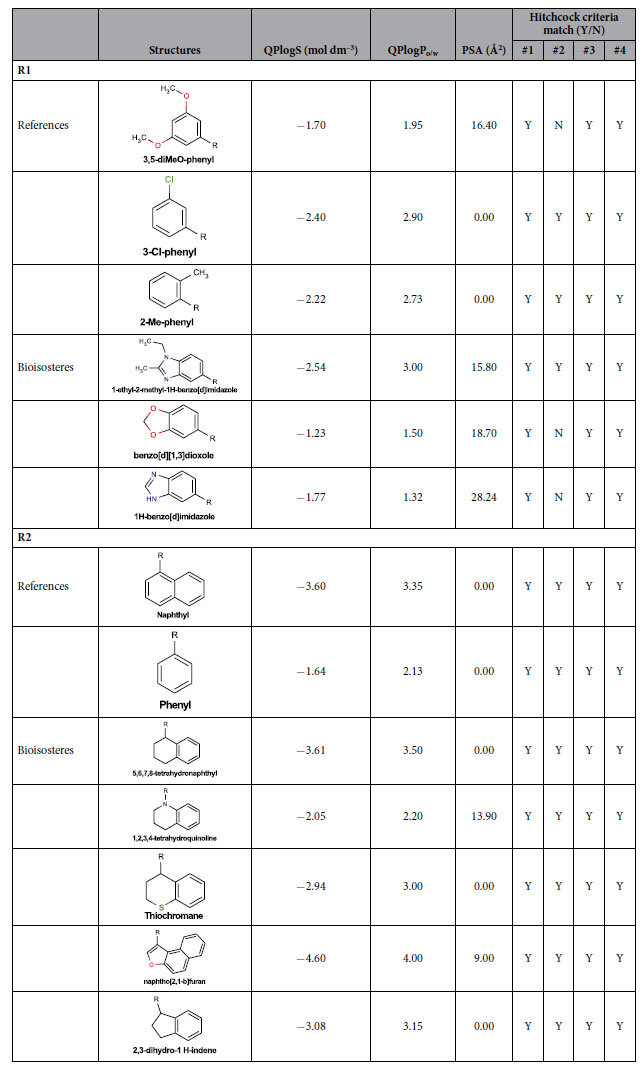
GPR139 bioisosteres with calculated solubility measures of QPlogS and QPlogP_o/w_.

Blood-brain barrier penetration has been estimated by calculating the polar surface area (PSA) and matching (Yes/No) of the Hitchcock criteria^18^:**#1** Sum of nitrogen and oxygen atoms (N + O) < 5, **#2** QPlogP – (N + O) > 0, **#3** PSA <90 Å2, **#4** MW < 450.

**Table 6:** Potencies and efficacies of 7c, 1a, _L_-Trp, _L_ -Phe, DL43, DL126, DL24, DL22, DL132 and DL130 as recorded in the IP-One HTRF^®^ assay.

Cmpd	EC_50_ (μM)	E_max_ (%)
7c (racemic)	0.49	120^(*)^
DL24	0.75	40
1a	1.05	100
DL132	4.16	77
DL22	4.18	69
DL130	4.98	19
DL126	5.62	38
DL43	9.57	65
L-Phe	4,454	95
L-Trp	~8,500	106^(**)^

Data represent the mean of at least three independent experiments performed in triplicates. Efficacies (**E**_**max**_) are relative to compound **1a**. (*)= The **E**_**max**_ for **7c** is based on the highest concentration tested (10 μM). (**)= The **E**_**max**_ for _**L**_**-Trp** is based on the highest concentration tested.(20 mM).
