# Supplementary material for: Novel Agonist Bioisosteres and Common Structure-Activity Relationships for The Orphan G Protein-Coupled Receptor GPR139
Source: Sci Rep. 2016 Nov 10;6:36681. doi: 10.1038/srep36681 (PMC5103216; doi:10.1038/srep36681)
Supplement: Supplementary Information [file srep36681-s1.pdf]

Supplementary Information

Novel Agonist Bioisosteres and Common Structure-Activity Relationships for The Orphan G Protein-Coupled Receptor GPR139

Mohamed A. Shehata, Anne C. Nøhr, Delphine Lissa, Christoph Bisig, Vignir Isberg, Kirsten B. Andersen, Kasper Harpsøe, Fredrik Björkling, Hans Bräuner-Osborne, and David E. Gloriam

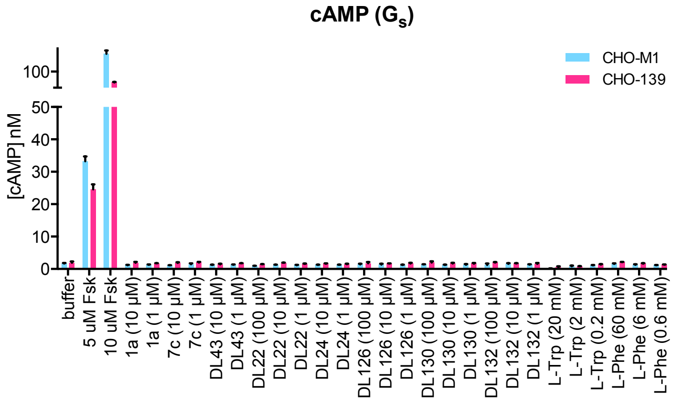

Supplementary Figure 1. cAMP dynamic 2 assay measuring G_s activation. 5,000 cells/well of CHO-139 (pink bars) or CHO-M1 (blue bars) cells are incubated with the indicated compounds for 30 min at room temperature in the presence of 50 µM IBMX. 5 µM and 10 µM forskolin (Fsk) serves as positive controls. The responses are one representative out of at two independent experiments performed in triplicates. Data points are means ± SD.

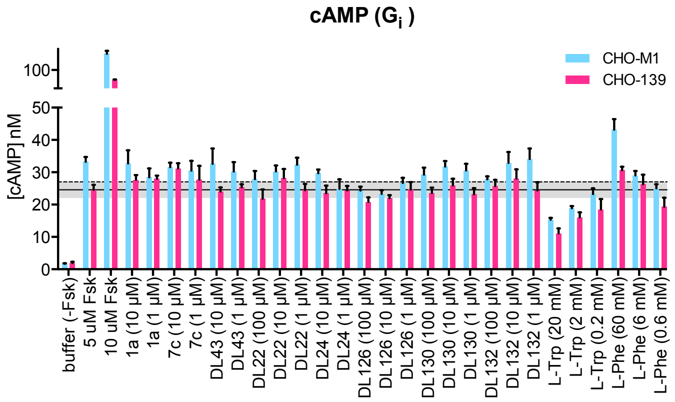

Supplementary Figure 2. cAMP dynamic 2 assay measuring G_i activation. 5,000 cells/well of CHO-139 (pink bars) or CHO-M1 (blue bars) cells are incubated with the indicated compounds for 30 min at room temperature in the presence of 50 µM IBMX and 5 uM Fsk (expect "buffer (-Fsk) where only 50 µM IBMX is present). The straight line indicate the level of cAMP upon activation with 5 uM Fsk, and the grey area ±10% of the response. The responses are one representative out of at two independent experiments performed in triplicates. Data points are means ± SD.

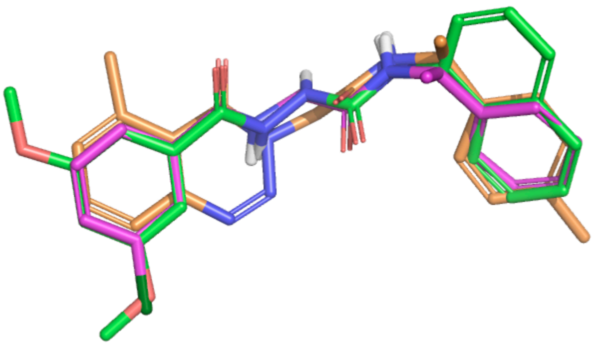

Supplementary Figure 3. Superposition of the lowest energy conformations of **1a** (Green), **7c** (Purple) and **39** (Orange), shows a very good alignment of the terminal phenyl and a 6-atom linker in which the last atom is part of a fused aromatic ring in the **1a-u** Lundbeck A/S compounds with the compounds from the other two series.

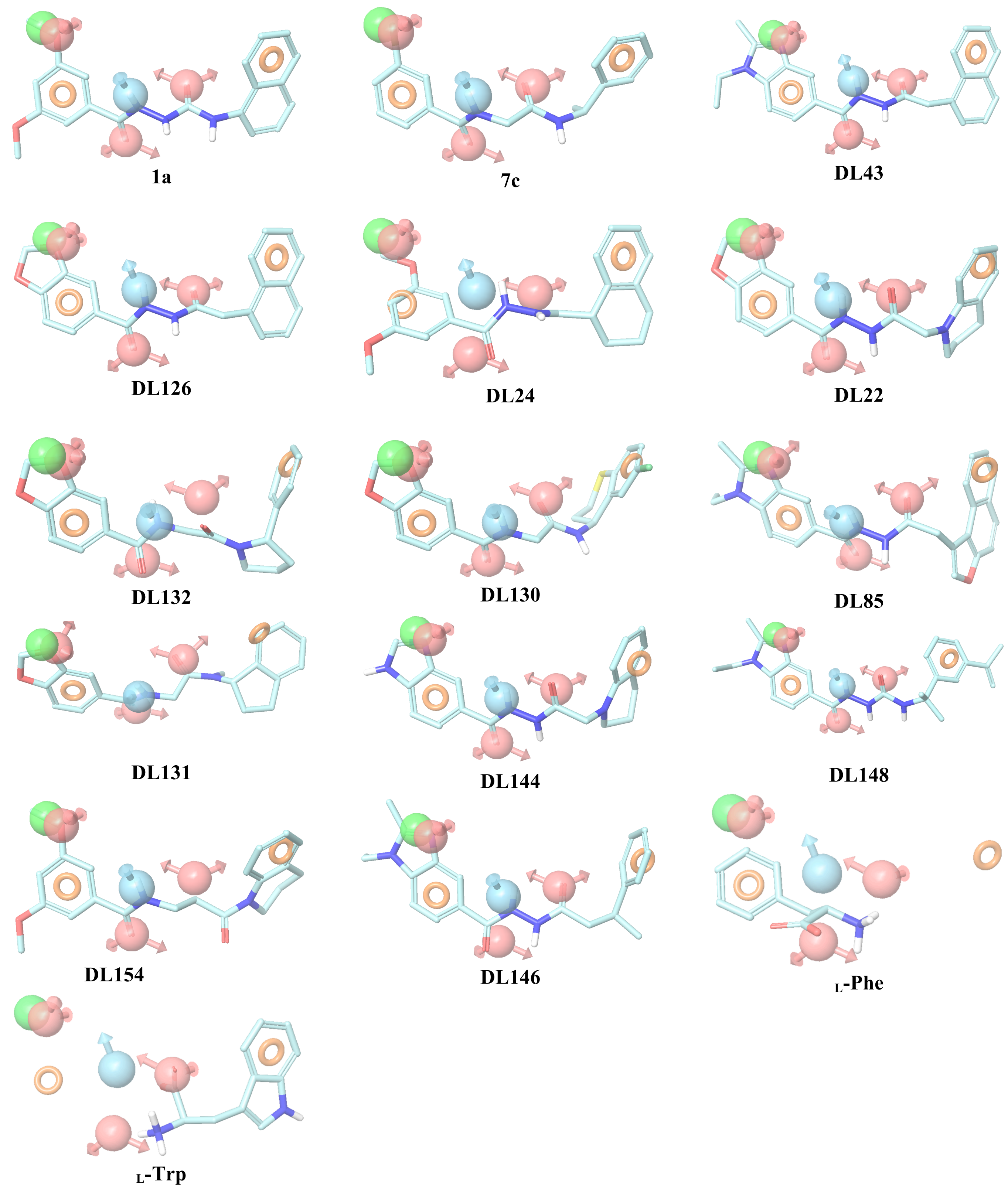

Supplementary Figure 4. Matching of our novel ligand bioisosteres to the common pharmacophore model. For clarity, labels and exclusion volumes are not displayed. The ligands show a good fit to the different features in the herein presented new model.

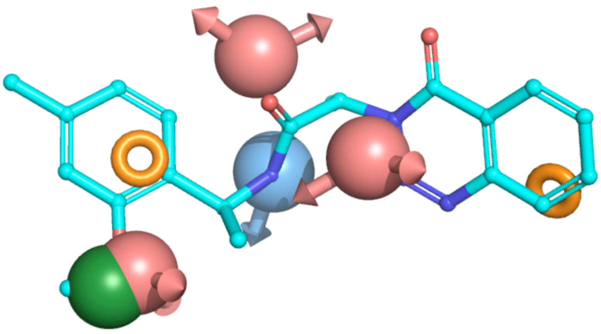

Supplementary Figure 5. Example of a horizontally flipped matching of a Hitchcock *et al.* compound (**50**) onto the suggested pharmacophore model.

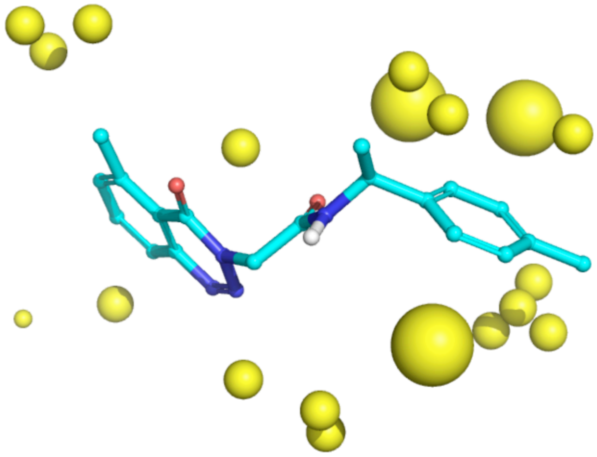

Supplementary Figure 6. Exclusion volumes are not in contradiction to the set by Hitchcock *et al.*

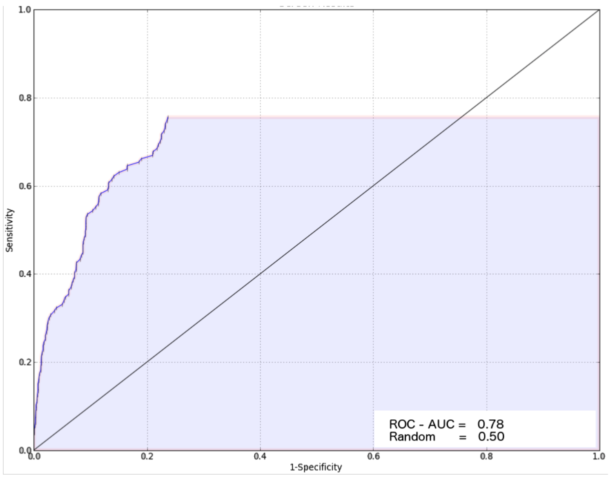

Supplementary Figure 7. ROC – Plot of the old pharmacophore performance on the same data set and decoys, shows a substantial difference between both models. The ROC-curve specifically shows the inability of the old pharmacophore to score all actives due to the lack of added new features in the new model as well as the corrected position of **R2** feature.

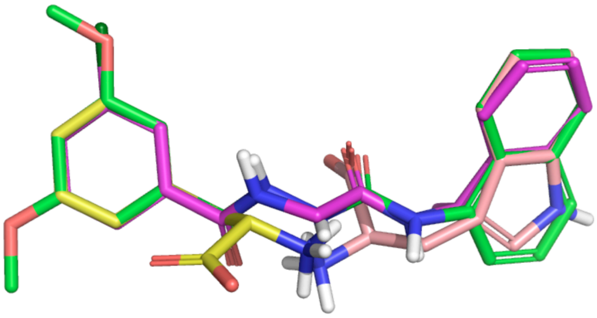

Supplementary Figure 8. Superposition of the amino acids L-Trp (pink) and L-Phe (yellow) displays a good alignment with the 1a (green) naphthyl and 7c (purple) benzyl, respectively, as well as 2 and 1 of the polar linker groups, respectively. This may suggest that the endogenous and surrogate ligands potentially share the same binding site, and that all four compounds are covered by the common pharmacophore model.

Supplementary Chart 1. Chemical structures of all tested analogs DL1–158.

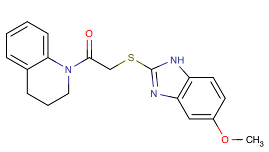
DL1

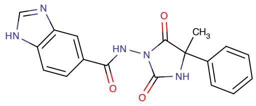
DL2

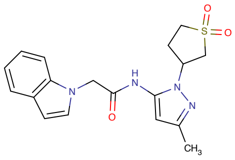
DL3

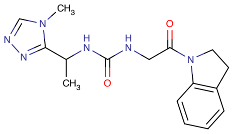
DL4

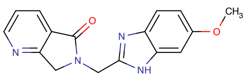
DL5

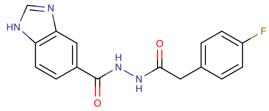
DL6

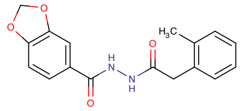
DL7

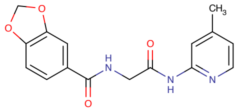
DL8

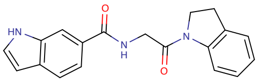
DL9

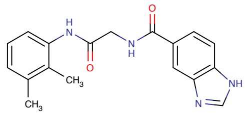
DL10

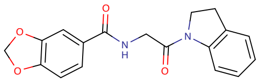
DL11

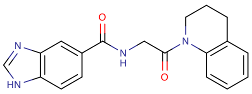
DL12

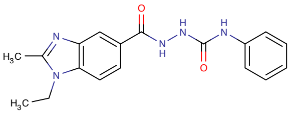
DL13

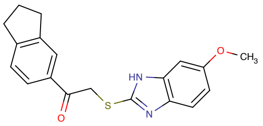
DL14

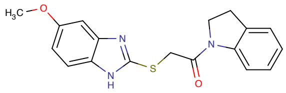
DL15

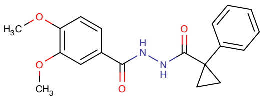
DL16

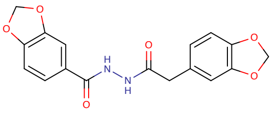
DL17

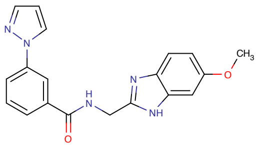
DL18

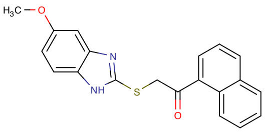
DL19

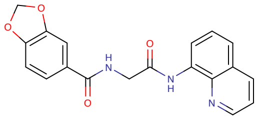
DL20

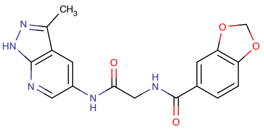
DL21

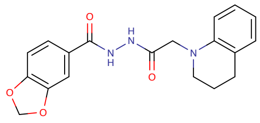
DL22

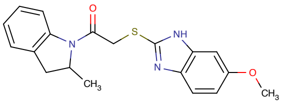
DL23

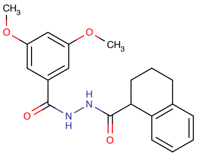
DL24

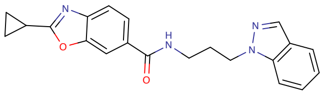
DL25

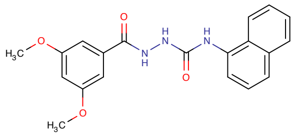
DL26 (1a)

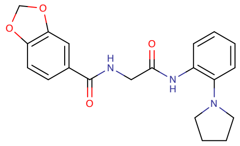
DL27

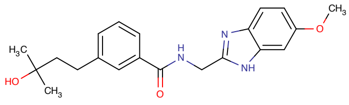
DL28

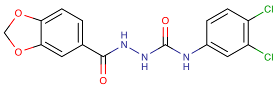
DL29

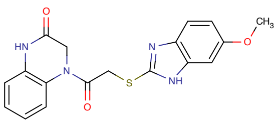
DL30

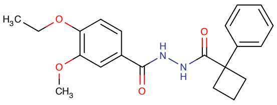
DL31

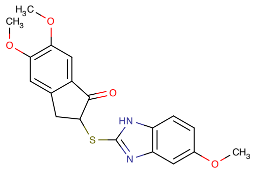
DL32

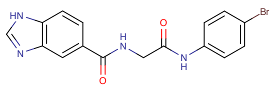

DL33

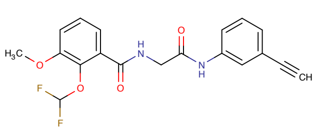

DL34

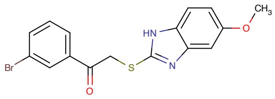

DL35

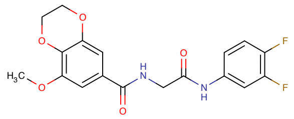

DL36

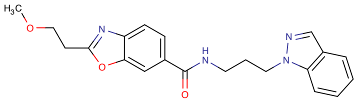

DL37

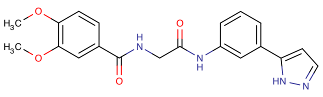

DL38

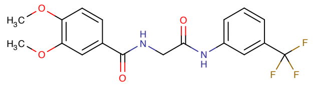

DL39

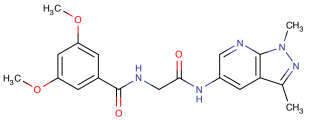

DL40

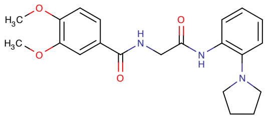

DL41

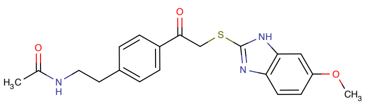

DL42

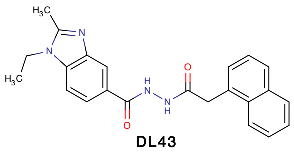

DL43

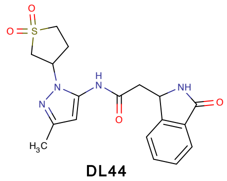

DL44

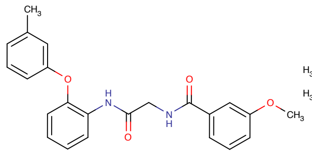

DL45

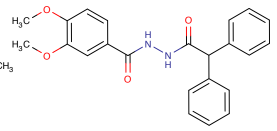

DL46

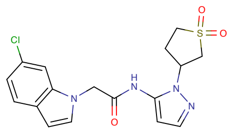

DL47

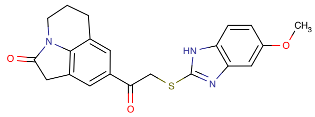

DL48

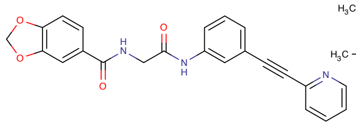

DL49

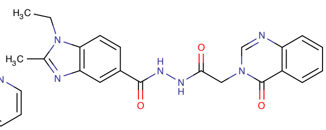

DL50

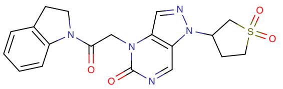

DL51

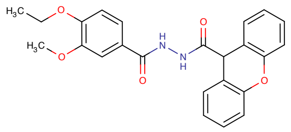

DL52

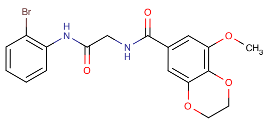

DL53

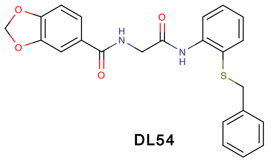

DL54

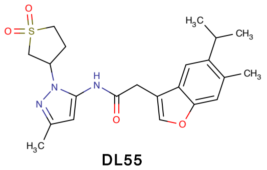

DL55

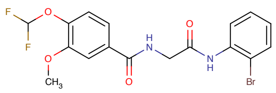

DL56

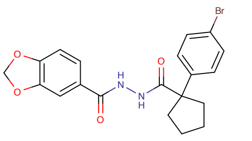

DL57

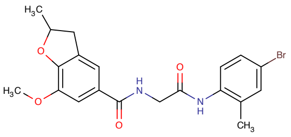

DL58

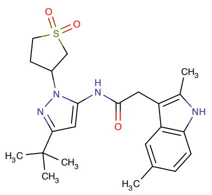

DL59

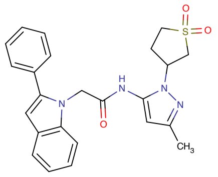

DL60

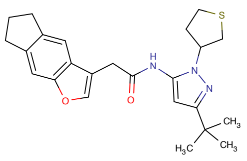

DL61

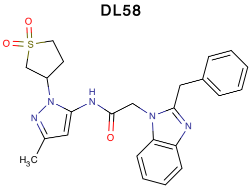

DL62

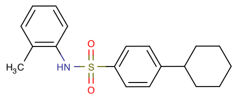

DL63 *

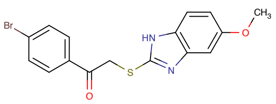

DL64

**Proposed antagonist identified by Hu et al..*

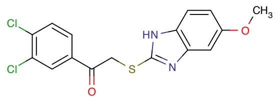

DL65

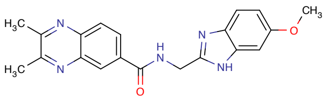

DL66

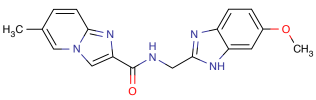

DL67

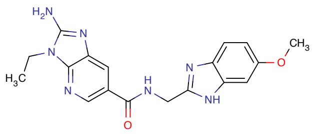

DL68

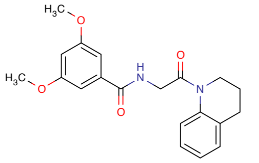

DL69

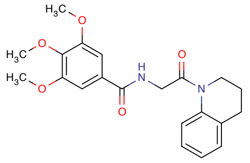

DL70 #

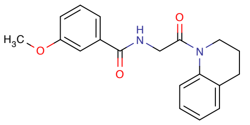

DL71

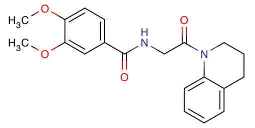

DL72

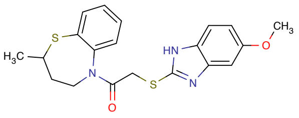

DL73

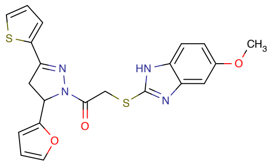

DL74

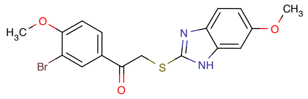

DL75

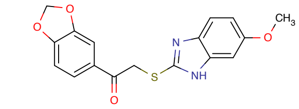

DL76

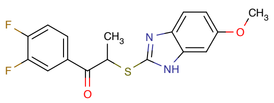

DL77

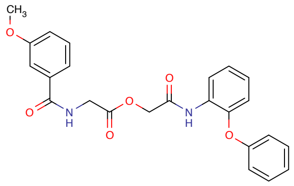

DL78

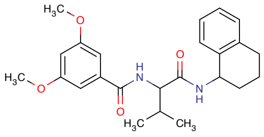

DL79

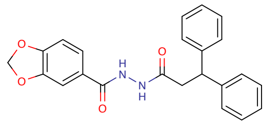

DL80

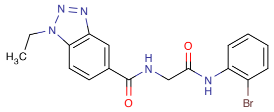

DL81

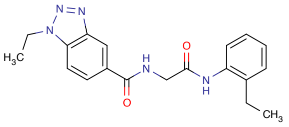

DL82

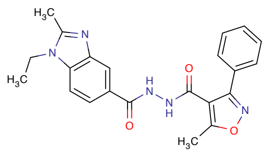

DL83 #

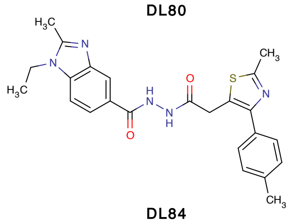

DL84

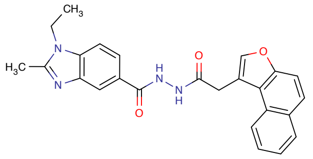

DL85

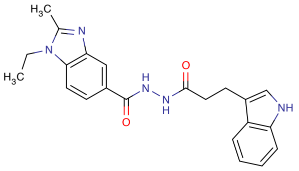

DL86

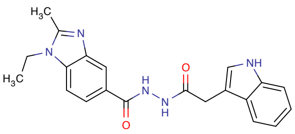

DL87

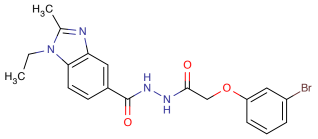

DL88

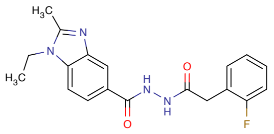

DL89 #

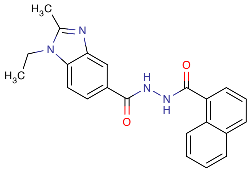

DL90

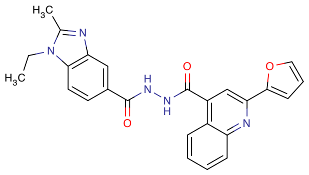

DL91

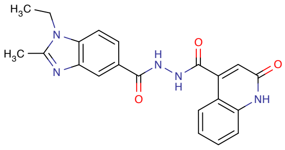

DL92

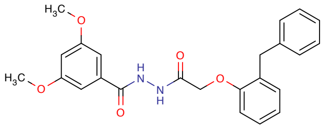

DL93

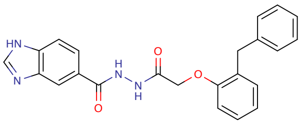

DL94

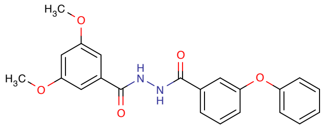

DL95

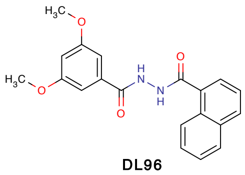

DL96

#*Very weak agonist (EC50 > 10 uM)*

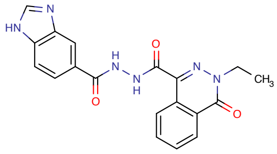

DL97

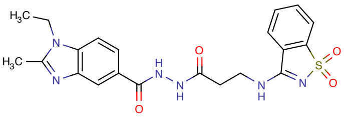

DL98

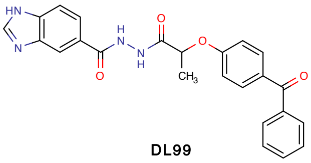

DL99

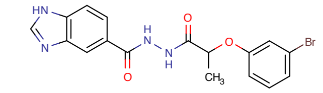

DL100 #

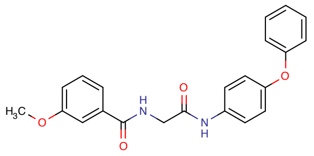

DL101

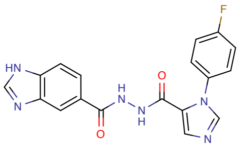

DL102

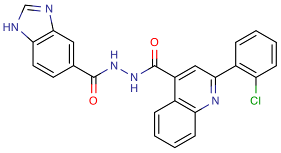

DL103

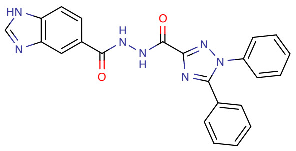

DL104

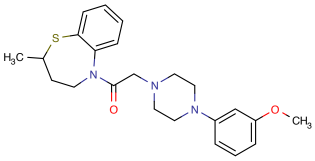

DL105

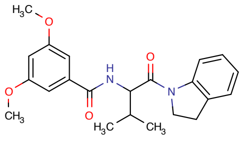

DL106

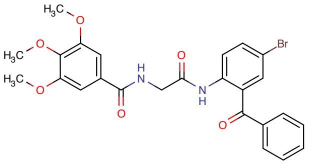

DL107

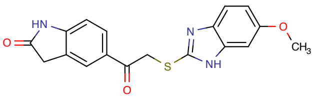

DL108

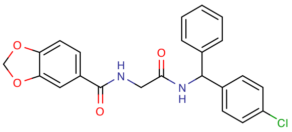

DL109

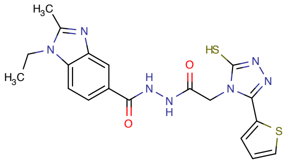

DL110

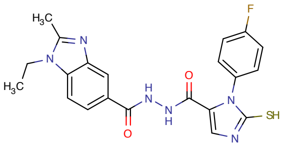

DL111

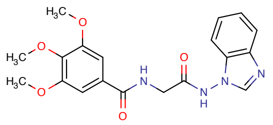

DL112

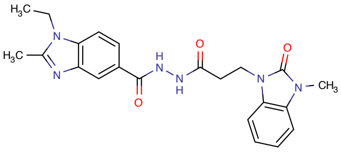

DL113

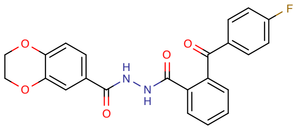

DL114

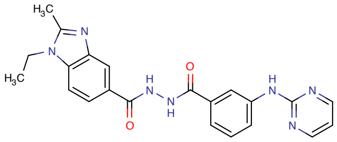

DL115

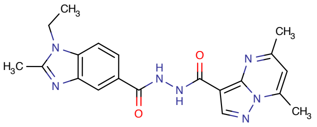

DL116

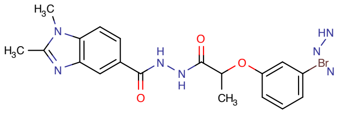

DL117

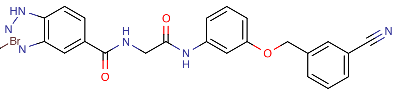

DL118

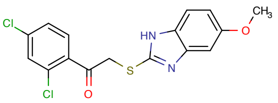

DL119

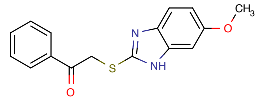

DL120

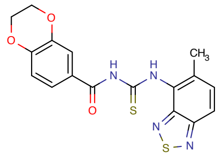

DL121

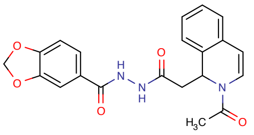

DL122

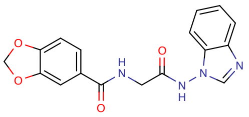

DL123

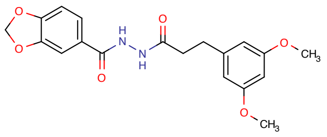

DL124

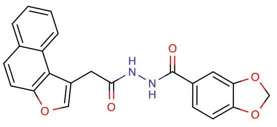

DL125

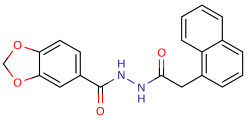

DL126

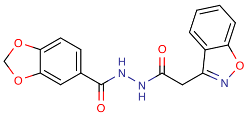

DL127

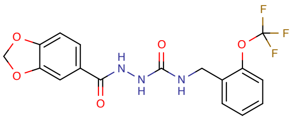

DL128

#*Very weak agonist (EC50 > 10 uM)*

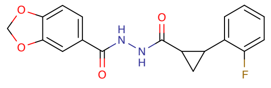
DL129

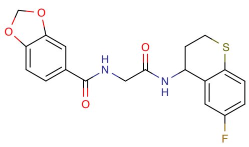
DL130

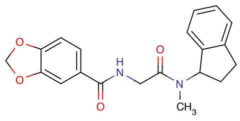
DL131

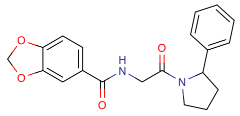
DL132

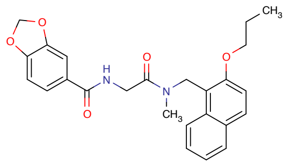
DL133

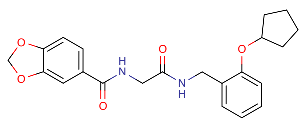
DL134

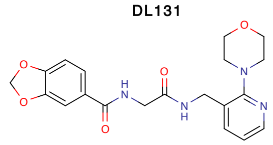
DL135

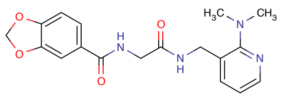
DL136

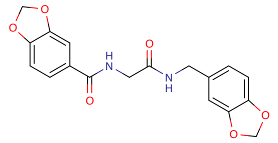
DL137 #

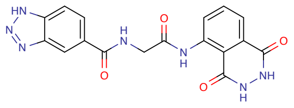
DL138

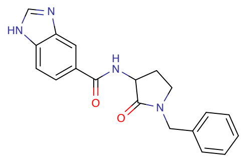
DL139

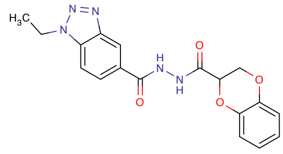
DL140

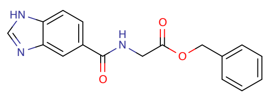
DL141

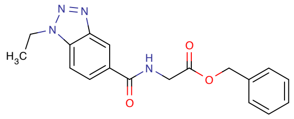
DL142

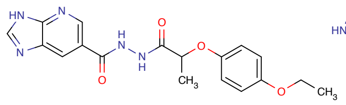
DL143

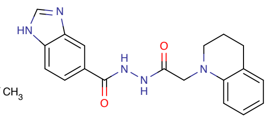
DL144

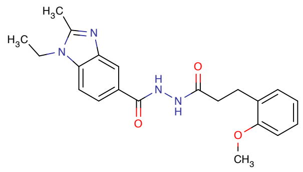
DL145

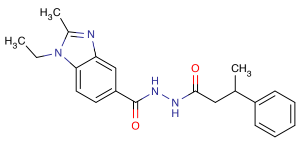
DL146

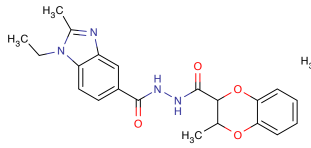
DL147

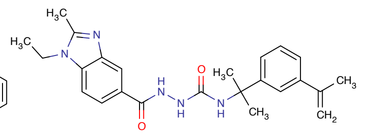
DL148

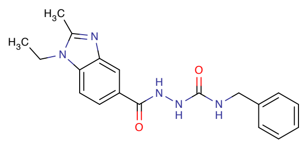
DL149

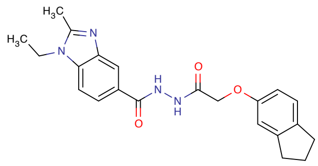
DL150

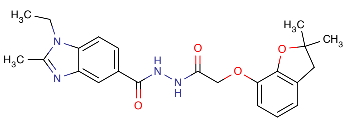
DL151

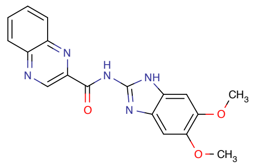
DL152

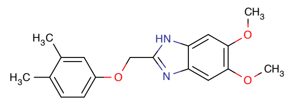
DL153

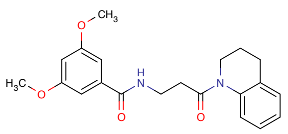
DL154

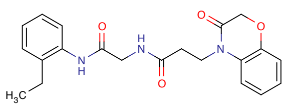
DL155

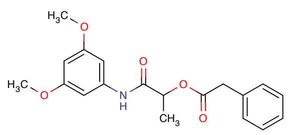
DL156

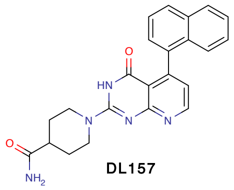
DL157

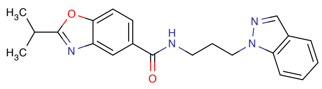
DL158

#Very weak agonist (EC50 > 10 uM
